# Outcomes of pregnancy with pulmonary hypertension: low risk or a false dawn?

**DOI:** 10.5830/CVJA-2023-014

**Published:** 2023-04-06

**Authors:** Onyedika J Ilonze

**Affiliations:** Division of Cardiovascular Medicine, Krannert Cardiovascular Research Center, Indiana University, Indianapolis, USA

I read with keen interest the article titled ‘A three-year audit of pregnancy outcomes in women with pulmonary hypertension admitted to the high-risk obstetric unit at Inkosi Albert Luthuli Central Hospital, KwaZulu-Natal, South Africa’ by Budhram et al.^1^

In this large, retrospective cohort study of 185 women, they reported on outcomes of women with pulmonary hypertension (PH) [World Health Organisation (WHO) group 1, n = 24, WHO group II, n = 151] with Doppler echocardiogram-derived pulmonary artery (PA) pressures. The results were similar to those of the ROPAC study,^2^ and the authors found that of that cohort, four women underwent elective termination of pregnancy and three had spontaneous miscarriages. The reported case-fatality rate at 42 days postpartum was 2.7%.

Cesarean section was the mode of delivery for almost 80% of patients. Gestational age and birth weight were inversely proportional to the severity of the PH. The proportion of low-birthweight (< 2 500 g) babies was also highest in patients with severe PH.

An important consideration is highlighted by this study. Traditionally data about morbidity associated with PH in pregnancy in the USA and Western Europe have focused on pulmonary artery hypertension (PAH)3 and not WHO group II PH. In this cohort, more than 75% of the cases were pregnancies in patients with WHO group II PH. Therefore, this study serves as an important addition to the literature.

Although 37.8% of the cohort were infected with human immunodeficiency virus (HIV), it remains unclear from the article whether HIV was the aetiology of the PH or they were WHO group I and II PH patients living with HIV. This distinction is important because HIV-associated PH is a rarer form of PH, for which robust data remain elusive. Data suggest that echocardiogram-derived PA pressures may correlate poorly with invasively derived PA pressures, but this method was used in the ROPAC study.^4^

Although this and recent trials^2^,^3^ have pointed to low mortality rates in pregnant PH patients, we would caution against excessive optimism for the following reasons. First, significant morbidity (high number of intensive care unit admissions in mothers and sub-optimal foetal outcomes – more pre-term delivery and low birthweight) remains in pregnancy-associated PH.

Second, most reported studies (including this one) have been done in centres with multidisciplinary teams (PH specialist, cardiothoracic surgery and anaesthesiologists and obstetricians who specialise in high-risk pregnancies) and advanced therapeutics, which may not be the norm in most of sub-Saharan Africa (SSA) where many patients with PH may reside.

Third, many young women with PH or PAH who deliver babies undergo precipitous decline in the postpartum period and die without extracorporeal membrane oxygenation or lung transplantation, which may not be readily available in SSA. Fourth, the retrospective nature of the study, despite its high sample size, may limit its generalisability, as was also acknowledged by the authors.

Nevertheless, this study gives us hope that adequate organisation of appropriate multidisciplinary teams in a resource-limited setting could achieve acceptable postpartum outcomes in this high-risk population. It also highlights the fact that most adverse events occur in the postpartum period when significant physiological changes occur, despite the patient apparently being ‘shepherded through a safe delivery’. A prospective validation of this study, if feasible, would add further to the existing literature on this topic. 
